# The Analysis of the Disease Spectrum in China

**DOI:** 10.1155/2014/601869

**Published:** 2014-05-22

**Authors:** Xin Zhang, Xiaoping Zhou, Xinyi Huang, Shumei Miao, Hongwei Shan, Shenqi Jing, Tao Shan, Jianjun Guo, Jianqiu Kou, Zhongmin Wang, Yun Liu

**Affiliations:** Department of Information, The First Affiliated Hospital, Nanjing Medical University, Nanjing 210029, China

## Abstract

Analysis of the related risks of disease provides a scientific basis for disease prevention and treatment, hospital management, and policy formulation by the changes in disease spectrum of patients in hospital. Retrospective analysis was made to the first diagnosis, age, gender, daily average cost of hospitalized patients, and other factors in the First Affiliated Hospital of Nanjing Medical University during 2006–2013. The top 4 cases were as follows: cardiovascular disease, malignant tumors, lung infections, and noninsulin dependent diabetes mellitus. By the age of disease analysis, we found a younger age trend of cardiovascular disease, and the age of onset of cancer or diabetes was somewhat postponed. The average daily cost of hospitalization and the average daily cost of the main noncommunicable diseases were both on the rise. Noncommunicable diseases occupy an increasingly important position in the constitution of the disease, and they caused an increasing medical burden. People should pay attention to health from the aspects of lifestyle changing. Hospitals should focus on building the appropriate discipline. On the other hand, an integrated government response is required to tackle key risks. Multiple interventions are needed to lower the burden of these diseases and to improve national health.

## 1. Introduction

Currently, the burden of global disease has changed greatly with the development of the national economy, the improvement of people's living standard, the deterioration of environment, the increasing pressure of work, the transformation of lifestyle, and other changes. The main diseases affecting human health have switched from acute and chronic infectious diseases to chronic noncommunicable diseases [1].

The study of the burden of disease provides an overall guidance for disease prevention and treatment, determines the level of medical technology and community medical demands, and is a reliable basis on which one can distribute the healthcare resources appropriately. The study of changes of disease spectrum has significance to find the disease regional characteristics, guide health policy, and solve the problem of shortage of medical resources. In view of what is mentioned above, we established a clinical database to conduct a further analysis of the changes of disease spectrum by the process of the clinical data of patients of the First Affiliated Hospital of Nanjing Medical University.

## 2. Materials and Methods 

### 2.1. Used Dataset

Our research objects are these patients who have been discharged from January 2006 to December 2013, and their data we used are from EMR (electronic medical record) and HIS (hospital information system) of the First Affiliated Hospital of Nanjing Medical University.

### 2.2. Methods

According to the primary diagnosis of hospitalized patients with ICD10 code [2], we established a clinical database to calculate the volume of patients of each disease and the percentage of the total cases of discharge, to rank the diseases and then to compare the percentages of different diseases in different years. There are many types of malignant tumors, so we coanalyzed all the malignant tumors and calculated various malignant tumors cases accounting for all malignant tumor cases proportion. The main types of cardiovascular diseases are coronary heart disease, cerebral infarction, hypertension, and arrhythmia, and we coanalyzed these four types of cardiovascular diseases. We made a count on the age and gender of the patients having these noncommunicable diseases. The age variable was categorized into three age groups: <40 years old, 41–60 years old, and >61 years old. We calculated the daily average hospitalization costs discharged each year and the daily average hospitalization costs of cardiovascular diseases and noninsulin dependent diabetes mellitus.

## 3. Results

### 3.1. General Review

There are 531718 discharges during 2006–2013. The average number is 66465 every year. Women account for 50.64%. The number of discharged patients increased year by year from 37105 in 2006 to 93040 in 2013.

### 3.2. The Change of Disease Sequence during 2006–2013

**Figure 1:**
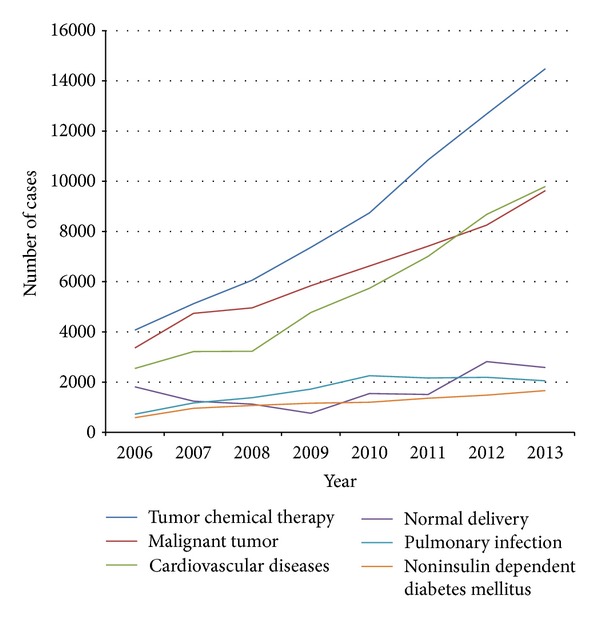
Trend of disease, 2006–2013. The horizontal axis represents year and the vertical axis represents discharges of each disease every year.

Table 1 provides an overall comparative view of disease sequence between 2006 and 2013. The top ten diseases in our hospital in 2013 were tumor chemical therapy, cardiovascular diseases, malignant tumor, normal delivery, other specified medical care (e.g., Z51.8 code in ICD-10. We can access the URL: “http://apps.who.int/classifications/apps/icd/icd10online2005/fr-icd.htm” or “http://apps.who.int/classifications/icd10/browse/2010/en#/Z51.8.” It was coded by WHO.), pulmonary infection, noninsulin dependent diabetes mellitus, cataract, chronic renal failure, and colon polyps. The trend of top six diseases is shown in Figure 1. The horizontal axis represents year and the vertical axis represents discharges of each disease every year.

Tumor chemical therapy is in the first place in disease sequence in all the 8 years and the number of discharges increased from 4072 in 2006 to 14481 in 2013. Since every patient of tumor chemical therapy needs to be treated in hospital several times, it is not the first disease. Malignant tumor is in the second place in disease sequence during 2006–2011 and in the third place during 2012-2013. It is a main disease of patients in our hospital.

Cardiovascular diseases were in the third place in disease sequence during 2006–2011 and have risen to the second place during 2012-2013. It is a main disease of elderly people in China. The most popular diseases are coronary heart disease, cerebral infarction, hypertension, and arrhythmia.

Pulmonary infection and noninsulin dependent diabetes mellitus both constituted the largest number for disease sequence.

### 3.3. Age Distribution of Cardiovascular Diseases

**Figure 2:**
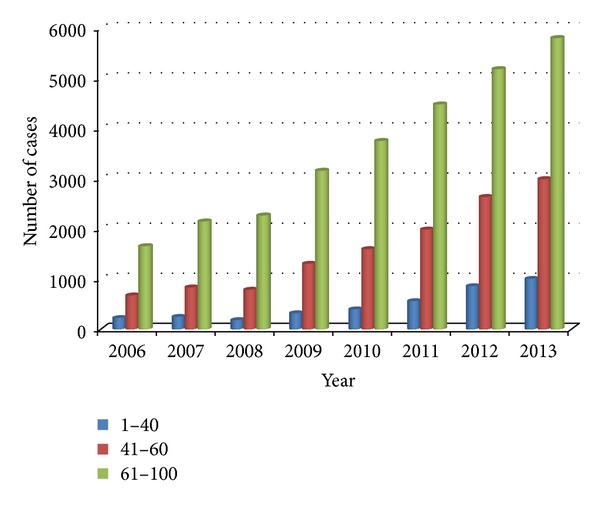
Age distribution of cardiovascular diseases. The horizontal axis represents year and the vertical axis represents discharges of each group of age every year.

The age distribution of cardiovascular diseases is shown in Figure 2. The horizontal axis represents year and the vertical axis represents discharges of each group of age every year. We noted that most patients are in the age group above 61 years. It is a common disease of the elderly. Recently, especially after 2009, patients in the age group below 40 years and 41–60 years increased year by year. There is no patient under 30 having coronary heart disease before 2009, while there are more and more patients under 30 having coronary heart disease since 2009. It provides the trend of younger age of cardiovascular diseases, which is coincident with the Global Burden of Diseases, Injuries, and Risk Factors Study 2010 (GBD 2010) by Yang et al. [3].

**Figure 3:**
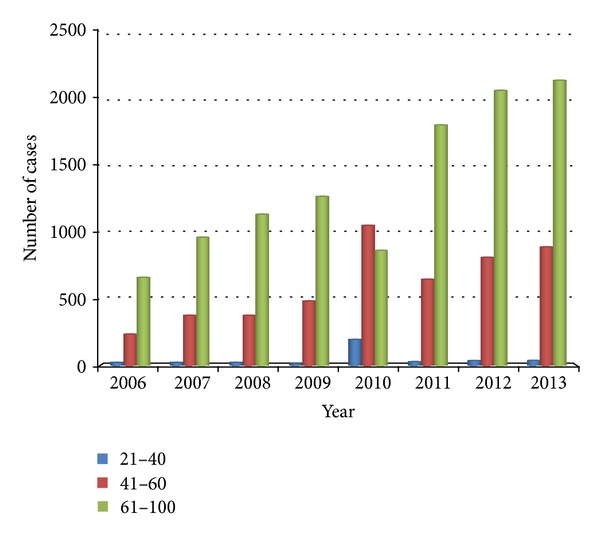
Age distribution of coronary heart diseases. The horizontal axis represents year and the vertical axis represents discharges of each group of age every year.

Coronary heart disease is a major cardiovascular disease, whose age distribution is shown in Figure 3. The horizontal axis represents year, and the vertical axis represents discharges of each group of age every year. We noted that most patients are in the age group above 61 years every year. By removing the extreme differences in individual year, it is shown that the volume of patients in the age group of 41–60 years has increased year by year, while that of patients in the age group above 60 years has reduced relatively. It provides the trend of younger age of coronary heart diseases and is consistent with the trend of younger age of cardiovascular diseases.

### 3.4. Age Distribution of Malignant Tumor

**Figure 4:**
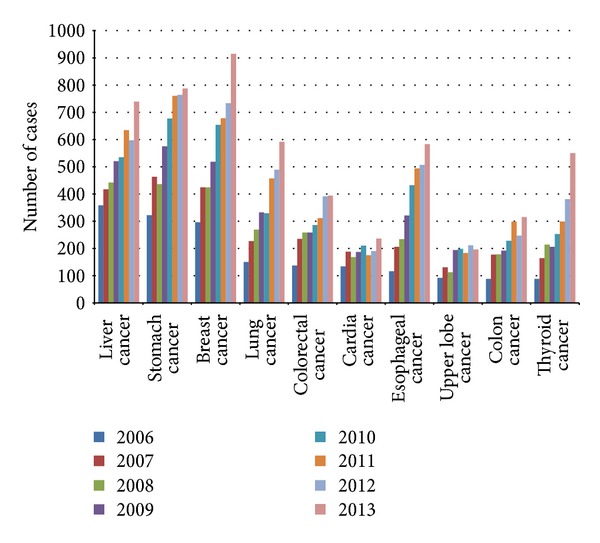
Trend of malignant tumor, 2006–2013. The horizontal axis represents the various cancers and the vertical axis represents the number of cancer patients in different years.

**Figure 5:**
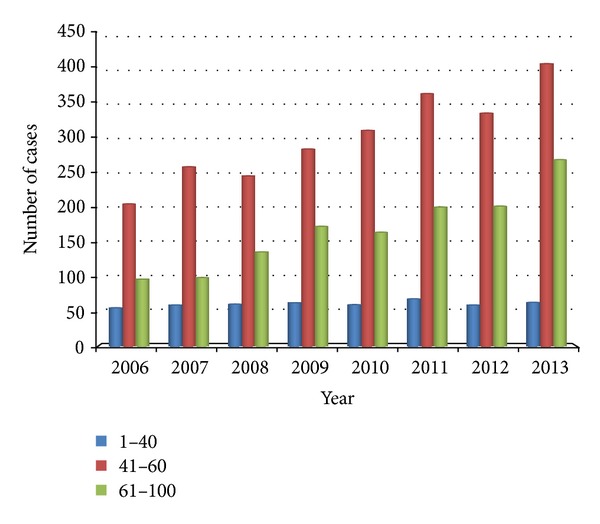
Age distribution of liver cancer. The horizontal axis represents year and the vertical axis represents the number of liver cancer patients in different age groups.

Figure 4 provides a status of malignant tumor between 2006 and 2013 of which the horizontal axis expresses the various cancers and the vertical axis expresses the number of cancer patients in different years. The top three cancers in sequence are stomach cancer, liver cancer, and breast cancer. An age distribution for liver cancer is shown in Figure 5 of which the horizontal axis expresses years and the vertical axis expresses the number of liver cancer patients in different age groups. We noted that, recently, especially after 2010, the volume of patients in the age group below 40 years and 41–60 years has decreased slowly while that of patients in the age group above 61 years increased dramatically.

### 3.5. Age Distribution of Noninsulin Dependent Diabetes Mellitus

**Figure 6:**
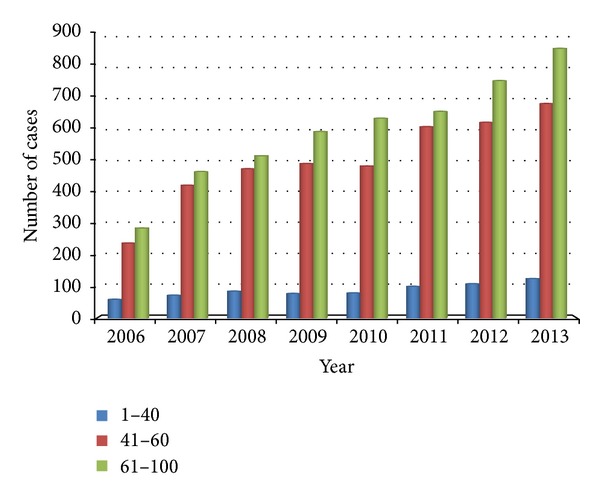
Age distribution of noninsulin dependent diabetes mellitus. The horizontal axis represents year and the vertical axis represents the volume of discharges of each group of age every year.

The age distribution of noninsulin dependent diabetes mellitus is shown in Figure 6 of which the horizontal axis represents year and the vertical axis represents discharges of each group of age every year. We noted that the volume of patients in the age group below 40 years has reduced from 9.97% in 2006 to 7.53% in 2013 and the volume of patients in the age group of 41–60 years has reduced from 41.07% in 2006 to 40.00% in 2013 in comparison with the fact that the volume of patients in the age group above 61 years has increased from 48.97% in 2006 to 51.48% in 2013.

### 3.6. The Cost of Major Noncommunicable Diseases Analysis

**Figure 7:**
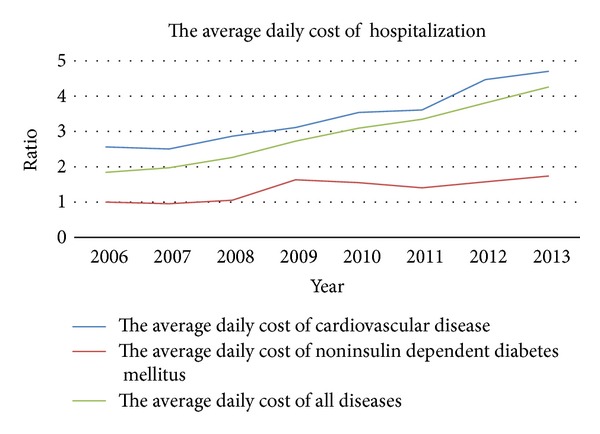
The average daily cost of hospitalization. The horizontal axis represents year and the vertical axis represents the ratio between the average daily cost of cardiovascular diseases or the average daily cost of noninsulin dependent diabetes mellitus each year and the average daily cost of noninsulin dependent diabetes mellitus in 2006.

**Figure 8:**
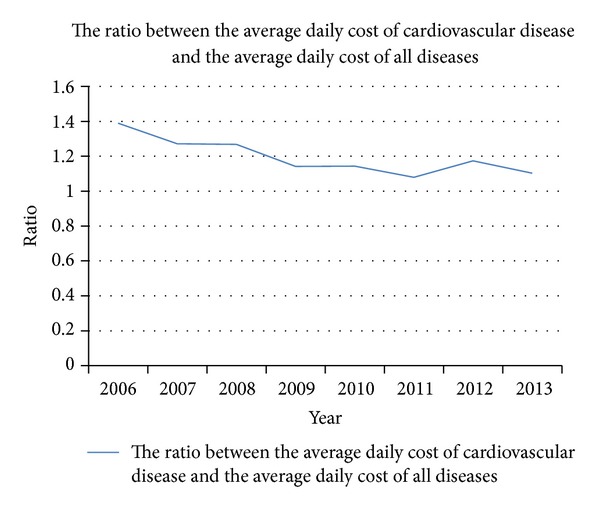
The ratio between the average daily cost of cardiovascular disease and the average daily cost of all diseases. The horizontal axis represents year and the vertical axis represents the ratio between the average daily cost of cardiovascular disease and the average daily cost of all diseases.

The average daily cost of hospitalization between 2006 and 2013 is shown in Figure 7. The horizontal axis represents year and the vertical axis represents the ratio between the average daily cost of cardiovascular diseases or the average daily cost of noninsulin dependent diabetes mellitus each year and the average daily cost of noninsulin dependent diabetes mellitus in 2006. The average daily cost has increased gradually year by year. The average daily cost of cardiovascular diseases and the average daily cost of noninsulin dependent diabetes mellitus have increased during recent years, which is consistent with the average daily cost of hospitalization. The ratio of average daily costs of cardiovascular diseases and average daily costs for all diseases in the same year is shown in Figure 8. The horizontal axis represents year and the vertical axis represents the ratio between the average daily cost of cardiovascular disease and the average daily cost of all diseases. We noted that there is a decreasing trend.

### 3.7. Gender Distribution Analysis

The gender distribution of cardiovascular diseases and noninsulin dependent diabetes mellitus is shown in Tables 2 and 3. We noted that men carry a higher burden of disease than women in both cardiovascular diseases and noninsulin dependent diabetes mellitus. Furthermore, there is an increasing trend of the men's higher burden in recent years.

## 4. Conclusion

The volume of patients in most of the major hospitals increased year by year with the improvements of people's living standard and healthcare demands. According to our data, the total volume of patients having been discharged from our hospital in 2013 was about 2.5 times as much as that in 2006 (Table 1). On one hand, it is because of extension of our hospital scale and establishment of the Group of Hospital; thus increasing sickbeds would also improve the ability of hospital medical services. On the other hand, the health policy “national basic medical insurance” covers more widely and benefits more people. We are getting closer to the goal: to assure that every citizen has equal access to affordable basic health care [4].

WHO (World Health organization) in Global Status Report on Noncommunicable Diseases 2010 has pointed out that noncommunicable diseases (NCDs) are the leading causes of death globally, killing more people each year than all other causes together [5]. Of the 57 million deaths that occurred globally in 2008, 36 million—almost two-thirds—were due to NCDs, comprising mainly cardiovascular diseases, cancers, diabetes, and chronic lung diseases [6]. The analysis results of disease composition in our hospital show that cardiovascular disease, cancer, diabetes, and lung infection are the most important diseases (Table 1, Figure 1), which are consistent with the above reports.

Cardiovascular diseases (CVDs) are the number one cause of death globally: more people die annually from CVDs than from any other cause [7, 8]. An estimated number of 17.3 million people died from CVDs in 2008, representing 30% of all global deaths. Of these deaths, an estimated number of 7.3 million were due to coronary heart disease and 6.2 million were due to stroke [9]. Our research found that, since 2012, cardiovascular disease has leapt to the first disease in our hospital (except chemotherapy for cancer treatment, Figure 1). In 2013, the volume of hospital discharged patients of cardiovascular disease is 10.52% of the total volume of hospital discharged patients (Table 1). Cardiovascular diseases, including coronary heart disease, have shown a trend of younger age (Figures 2 and 3). Therefore, the improvement of the prevention and treatment for cardiovascular disease cannot be delayed [10, 11].

Cancer is another leading cause of death worldwide, accounting for 8.2 million deaths in 2012 [12] (IARC). Freddie Bray predicts an increase in the incidence of all-cancer cases from 12.7 million new cases in 2008 to 22.2 million by 2030 [13]. Malignant tumors have been in the first place in the sequence of disease in our hospital during 2006 to 2011 and in the second place during 2012 to 2013 (Figure 1). In 2013, the discharged patients of malignant tumors from our hospital are 10.35% of the total discharged patients from our hospital (Table 1). Lung, liver, stomach, colorectal, and breast cancers cause the most cancer deaths each year [14, 15]. In 2013, the volume of breast cancer discharged patients is 9.5% of the total volume of hospital discharged patients, which is in the first place, while the volume of stomach cancer, liver cancer, and lung cancer discharged patients presents 8.17%, 7.67%, and 6.15% of the total volume of hospital discharged patients (Figure 4). Ageing is a fundamental factor for the development of cancer. The incidence of cancer rises dramatically with age, most likely due to a build-up of risks for specific cancers that increase with age. The overall risk accumulation is combined with the tendency for cellular repair mechanisms to be less effective as a person grows older [14]. In the age distribution of liver cancer in our hospital, the age group below 40 years has the least discharged patients; the volume in this group is 8.53% of the total volume of discharged patients of liver cancer. The age group of 41–60 years and the age group above 61 years both have a lot of patients. The volumes in these groups are 55.07% and 36.40% of the volume of total discharged patients of liver cancer, respectively (Figure 5).

Liver cancer is the second cause of death from cancer worldwide. The prognosis for liver cancer is very poor (overall ratio of mortality to incidence of 0.95) [12]. Our data shows that the survival time of liver cancer is 0.69 year. The number may be lower than the fact because of the lack of follow-up data. Most of liver cancer patients find their cancer too late, because they have no early symptom. So the high risk group should check regularly.

The findings of Ravi Prakash Upadhyay pointed out a high burden of diabetes and prediabetes [16]. The prevalence of diabetes in the United States has nearly tripled in the past couple of decades. From 1990 to 2010, the prevalence of diabetes in those aged >18 years increased from 6.6 million in 1990 to 20.7 million in 2010 [17]. The volume of discharges of noninsulin dependent diabetes mellitus increased from 582 in 2006 to 1661 in 2013; it is 2.85 times. Programs should be implemented to educate the community regarding the disease, its signs/symptoms, importance of early detection, and treatment along with ensuring availability of trained staff and well equipped health facilities.

The epidemic of these noncommunicable diseases is being driven by powerful forces now touching every region of the world: demographic ageing, rapid unplanned urbanization, and the globalization of unhealthy lifestyles. Gonghuan Yang pointed that dietary risk factors, high blood pressure, and tobacco exposure are the risk factors that constituted the largest number of attributable DALYs (disability-adjusted life years) in China [3]. Lim et al. pointed that, in 2010, the three leading risk factors for global disease burden were high blood pressure, tobacco smoking including second-hand smoke, and household air pollution from solid fuels. Dietary risk factors (diets low in fruits and those high in sodium) and physical inactivity are also important [18]. Nowadays, a menu of options are set out for addressing these diseases through both population-wide interventions, largely aimed at prevention, and individual interventions, aimed at early detection and treatment that can reduce progression to severe and costly illness and complications [5]. In our research findings, the ratio that the noninsulin dependent diabetes mellitus patients aged <40 accounting for the diabetes patients reduces from 9.97% in 2006 to 7.53% in 2013 (Figure 6) and reveals that the age of onset of noninsulin dependent diabetes mellitus is postponed due to these effective intervention solutions mentioned above possibly. The rise of inpatient expenditures is caused by the rise of hospital costs, the rise of labor costs, the emerging of new medic methods with the development of science and technology, and the rising demand for health care jointly. The literature shows that health care spending has grown faster than that of income during 1993 to 2003 [4]. According to our analysis of data, the mean inpatient expenditures rise increasingly year to year, that the average cost of 2013 was 2.3 times as much as that of 2006 (Figure 2).

WHO pointed that the costs of health-care systems from noncommunicable diseases are high and increasing [5]. In recent years, the average daily hospitalization costs of cardiovascular disease and noninsulin dependent diabetes mellitus in our hospital are both rising. The average daily hospitalization cost of cardiovascular disease in 2013 was 1.83 times as much as that in 2006. The average daily hospitalization cost of noninsulin dependent diabetes mellitus in 2013 was 1.74 times as much as that in 2006 (Table 2). The average daily hospitalization cost of cardiovascular disease is much higher than the average daily hospitalization cost of all the diseases every year. The average daily hospitalization cost of cardiovascular disease was 1.39 times as much as that of the average daily hospitalization cost of all the diseases in 2006. It showed that the costs from noncommunicable diseases are high. Comparing the ratio between the average daily hospitalization cost of cardiovascular disease and the average daily hospitalization cost, we found that the ratio has decreased. It may show that people paid more attention to the cardiovascular disease in recent years. Vigorous propaganda on cardiovascular disease, preventing it through lifestyle changing, and all the other various interventions could improve the disease burden of cardiovascular disease [5].

These noncommunicable diseases are the focus of hospital medical operation management. Hospitals should construct the appropriate discipline in accordance with the rules and characteristics of the disease, adjust settings or optimize related specialties, and strengthen discipline construction.

On the other hand, people often have the point of view on noncommunicable diseases as problems solely resulting from harmful individual behaviours and lifestyle choices and blame victims. The influence of socioeconomic circumstances on risk and vulnerability to noncommunicable diseases and the impact of health-damaging policies are not always fully understood. Reduction of population exposures from poor diet, high blood pressure, tobacco use, cholesterol, and fasting blood glucose are public policy priorities for China, as are the control of ambient and household air pollution [5]. These changes will require an integrated government response to improve primary care and undertake required multisectoral action to tackle key risks.

## Figures and Tables

**Table 1:** Cases and sequence of diseases leaving our hospital each year, 2006–2013.

Diagnosis	Year															
	2013		2012		2011		2010		2009		2008		2007		2006	
	Number of cases (%)	Pos.	Number of cases (%)	Pos.	Number of cases (%)	Pos.	Number of cases (%)	Pos.	Number of cases (%)	Pos.	Number of cases (%)	Pos.	Number of cases (%)	Pos.	Number of cases (%)	Pos.
Total	93040		87273		77621		69301		62205		53903		51270		37105	
Tumor chemical therapy	14481 (15.56)	1	12684 (14.53)	1	10849 (13.98)	1	8739 (12.61)	1	7366 (11.84)	1	6061 (11.24)	1	5122 (9.99)	1	4072 (10.97)	1
Cardiovascular diseases	9790 (10.52)	2	8682 (9.95)	2	7011 (9.03)	3	5739 (8.28)	3	4774 (7.67)	3	3226 (5.98)	3	3217 (6.27)	3	2546 (6.86)	3
Malignant tumor	9629 (10.35)	3	8256 (9.46)	3	7418 (9.56)	2	6624 (9.56)	2	5842 (9.39)	2	4960 (9.20)	2	4742 (9.25)	2	3363 (9.06)	2
Normal delivery	2580 (2.77)	4	2817 (3.23)	4	1510 (1.95)	6	1547 (2.23)	5	758 (1.22)	7	1123 (2.08)	5	1243 (2.42)	4	1813 (4.89)	4
Other specified medical care	2054 (2.21)	5	1855 (2.13)	6	1570 (2.02)	5	1220 (1.76)	6	1034 (1.66)	6	753 (1.40)	7	578 (1.13)	7	506 (1.36)	8
Pulmonary infection	2053 (2.21)	6	2193 (2.51)	5	2164 (2.79)	4	2255 (3.25)	4	1720 (2.77)	4	1380 (2.56)	4	1171 (2.28)	5	725 (1.95)	5
Noninsulin dependent diabetes Mellitus	1661 (1.79)	7	1480 (1.70)	7	1361 (1.75)	7	1198 (1.73)	7	1160 (1.86)	5	1071 (1.99)	6	959 (1.87)	6	582 (1.57)	6
Cataract	1080 (1.16)	8	1153 (1.32)	8	736 (0.95)	8	513 (0.74)	11	482 (0.77)	10	474 (0.86)	9	330 (0.64)	14	271 (0.73)	10
Chronic Renal Failure	908 (0.98)	9	907 (1.04)	9	599 (0.77)	10	584 (0.84)	8	568 (0.91)	8	409 (0.76)	11	355 (0.69)	13	267 (0.72)	11
Colon Polyps	854 (0.92)	10	739 (0.85)	10	343 (0.44)	17	214 (0.31)	35	235 (0.38)	28	118 (0.22)	68	113 (0.22)	77	70 (0.19)	73

**Table 2:** Gender distribution of cardiovascular diseases, 2006–2013.

Gender		2006	2007	2008	2009	2010	2011	2012	2013
Male	Number of cases	1640	2084	2135	3165	3628	4282	5254	5991
	Percentage (%)	64.4	64.8	66.2	66.3	63.2	61.1	60.5	61.2

Female	Number of cases	906	1133	1091	1609	2111	2729	3428	3799
	Percentage (%)	35.6	35.2	33.8	33.7	36.8	38.9	39.5	38.8

Total		2546	3217	3226	4774	5739	7011	8682	9790

**Table 3:** Gender distribution of noninsulin dependent diabetes mellitus, 2006–2013.

Gender		2006	2007	2008	2009	2010	2011	2012	2013
Male	Number of cases	337	559	600	655	718	835	918	1053
	Percentage (%)	57.9	58.3	56.0	56.5	59.9	61.4	62.0	63.4

Female	Number of cases	245	400	471	505	480	526	562	608
	Percentage (%)	42.1	41.7	44.0	43.5	40.1	38.6	38.0	36.6

Total		582	959	1071	1160	1198	1361	1480	1661
